# Role of Aquaporins in Determining Carbon and Nitrogen Status in Higher Plants

**DOI:** 10.3390/ijms19010035

**Published:** 2018-01-15

**Authors:** Limin Gao, Zhifeng Lu, Lei Ding, Junjie Guo, Min Wang, Ning Ling, Shiwei Guo, Qirong Shen

**Affiliations:** 1 Jiangsu Provincial Key Lab for Organic Solid Waste Utilization, National Engineering Research Center for Organic-based Fertilizers, Jiangsu Collaborative Innovation Center for Solid Organic Waste Resource Utilization, Nanjing Agricultural University, Nanjing 210095, China; limingao@njau.edu.cn (L.G.); luzhifeng@njau.edu.cn (Z.L.); 2014203030@njau.edu.cn (J.G.); minwang@njau.edu.cn (M.W.); nling@njau.edu.cn (N.L.); shenqirong@njau.edu.cn (Q.S.); 2 Institut des Sciences de la Vie, Université Catholique de Louvain, Louvain-la-Neuve B-1348, Belgium; lei.ding@uclouvain.be

**Keywords:** aquaporins, carbon, nitrogen, transport, uptake

## Abstract

Aquaporins (AQPs) are integral membrane proteins facilitating the transport of water and some small neutral molecules across cell membranes. In past years, much effort has been made to reveal the location of AQPs as well as their function in water transport, photosynthetic processes, and stress responses in higher plants. In the present review, we paid attention to the character of AQPs in determining carbon and nitrogen status. The role of AQPs during photosynthesis is characterized as its function in transporting water and CO_2_ across the membrane of chloroplast and thylakoid; recalculated results from published studies showed that over-expression of AQPs contributed to 25% and 50% increases in stomatal conductance (*g_s_*) and mesophyll conductance (*g_m_*), respectively. The nitrogen status in plants is regulated by AQPs through their effect on water flow as well as urea and NH_4_^+^ uptake, and the potential role of AQPs in alleviating ammonium toxicity is discussed. At the same time, root and/or shoot AQP expression is quite dependent on both N supply amounts and forms. Future research directions concerning the function of AQPs in regulating plant carbon and nitrogen status as well as C/N balance are also highlighted.

## 1. Introduction

As a member of the major intrinsic protein (MIP) families, Aquaporins (AQPs) have been demonstrated to be integral membrane proteins that function in facilitating water transport across biological membranes [1,2]. Aquaporins in plant plasma and intracellular membranes are classified to five subgroups according to phylogenetic analyses. Plasma membrane intrinsic proteins (PIPs, with two phylogenic subgroups, PIP1 and PIP2) and tonoplast intrinsic proteins (TIPs) are considered to be the most abundant aquaporins in the plasma membrane and vacuolar membrane, respectively [3]. Nodulin-26-like intrinsic proteins (NIPs) are expressed in the peribacteroid membrane of N_2_-fixing symbiotic root nodules, but they are also present in the plasma membrane or the endoplasmic reticulum of the nonlegume plant species [4]. Small basic intrinsic proteins (SIPs) comprise only 2–3 isoforms and are mostly localized in the endoplasmic reticulum [5]. An uncharacterized AQP subfamily was recently identified in the genomes of lower plants and dicots and named X intrinsic proteins (XIPs) [6]. Some lower plant species have acquired additional aquaporins, including GlpF-like intrinsic protein (GIPs) and hybrid intrinsic proteins (HIPs). The function of AQPs was firstly reported as water channel proteins; later on, functional expression of plant AQPs in *Xenopus oocytes* and/or yeast cells pointed to a great diversity of substrates, including CO_2_, NH_3_, urea, glycerol, H_2_O_2_, boric acid, and silicic acid, etc. [7,8,9,10]. The diversity of the AQP substrates illustrates their complex integrated roles in regulating plant growth and metabolic processes, which have been widely reviewed in past years [11,12,13,14,15].

Efficient assimilation of carbon (C) and nitrogen (N) and the balance between them are essential for optimal plant growth, productivity, and yield [16]. Previously, different strategies have been found to associate with the C–N ratio in higher plants. For instance, nitrate supply leads to rapid changes in the levels of a wide range of transcripts encoding enzymes in N and carbon metabolism, and is performed in order to achieve a better balance between C and N [16]; it also leads to increased allocation of biomass to shoots and increased shoot density, which are also considered important for sustaining the C–N ratio [17,18]. Since AQPs have established roles in transporting CO_2_, urea, and NH_3_, it is reasonable to speculate that AQPs are involved in carbon and nitrogen metabolism, a role rarely considered. This review aims to examine the role of aquaporins in regulating C and N status. In addition, the regulation of AQP expression by nitrogen is also discussed.

## 2. The Role of AQPs in Regulating Carbon Status

**Figure 1:**
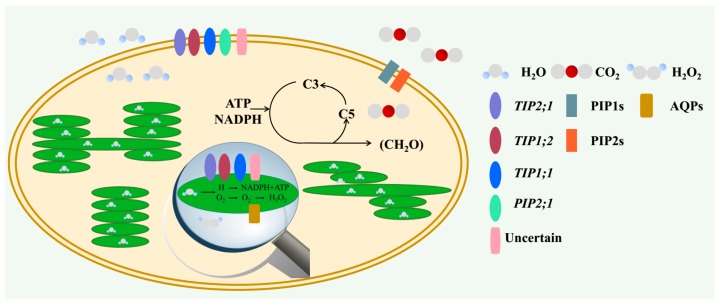
AQPs (Aquaporins) located in the chloroplast and thylakoid membrane are associated with the facilitation of H_2_O, CO_2_, and H_2_O_2_. The figure illustrates the variety of transport functions achieved by aquaporins in the chloroplast, the arrows presented the photosynthesis process briefly. The different aquaporin subclasses and functions are identified at the right of the illustration in distinct colors and shapes. The TIPs (tonoplast intrinsic proteins) were demonstrated to be located in the thylakoid and chloroplast and facilitate the transportation of H_2_O in the chloroplast, and the *PIP2;1* were recently identified in the *Arabidopsis* envelope fraction. Still, some uncertain AQPs, such as *PIP1;2*, *PIP1;3*, *PIP2;4*, and *PIP2;7* were also detected in envelope membrane preparations in the chloroplast, though they were considered as contaminants—this needs to be clarified in the future. The PIP1s (plasma membrane intrinsic proteins (PIPs), subgroup 1) in *Arabidopsis thaliana*, tobacco, and maize, and the PIP2s (PIPs, subgroup 2) in rice plant are considered as CO_2_ facilitators. Moreover, AQPs facilitate transmembrane diffusion of H_2_O_2_ with a heterologous expression system, but the related isoforms and evidence need to be studied in the future.

As AQPs commonly transport water, their role in regulating photosynthesis has been studied. In the thylakoid membrane, H_2_O is reduced to O_2_, and is paralleled with the conversion of light energy to chemical energy. The chemical energy generated during the light reaction is essential for the incorporation of CO_2_ to carbohydrate, which takes place in chloroplast stroma. Aquaporins are present in chloroplast (Figure 1) and the functions of them in the C status are summarized in this part.

### 2.1. Water Transport Across the Membrane of Chloroplast and Thylakoid

In the chloroplast, the extraction of electrons from water and transport through the electron transfer chain to NADP^+^ drives the fixation of CO_2_ into carbohydrates, which takes place in the thylakoid. How does the water make it into the thylakoid? The two possible routes are free diffusion or facilitated diffusion by AQPs. By quantifying the amount of water consumed during photosynthetic reactions and the amounts available inside the chloroplast stroma and thylakoid lumen, it was concluded that the amount of water present within the stroma and lumen were 2.5- and 30-fold lower than the amount of water needed to perform photosynthetic water oxidation per day [19]. Hence, a large amount of water transport across the thylakoid membrane was demanded to ensure high water oxidation rates. It was calculated that the ratio of diffusional permeability (*P_d_*) to osmotic permeability (*P_f_*) was less than 1, which indicates the presence of water channels in biological membranes [19,20,21].

Using mass-spectrometry-based proteomics, Zybailov et al. [22] detected the location of *TIP2;1* on the lumenal site of the thylakoid in *Arabidopsis thaliana*, and *TIP2;1* and *TIP1;2* in the thylakoid fraction and chloroplast membranes were also detected [23]. Besides *TIP1;1*, peptides for some PIPs (*PIP1;2*, *PIP1;3*, *PIP2;4*, and *PIP2;7*), were also detected in envelope membrane preparations, indicating the presence of aquaporins in the chloroplast; however, these AQPs cannot be ruled out as contaminants in the study by Ferro et al. [23]. Recently, *TIP1;1* and *PIP2;1* were identified by mass spectrometry in the *Arabidopsis* envelope fraction by Simm et al. [24]. In *Nicotiana tabacum*, it was also shown that both the plasma membrane and inner chloroplast membranes contained aquaporin *NtAQP1*, even though its expression in *Xenopus oocytes* allows only very low water transport rates [25]. Taken together, both the theoretical calculations and the presence of AQPs in cellular membranes supported the hypothesis that AQPs participate in water transport across the thylakoid membranes [19,26]. However, there is still little direct evidence to demonstrate the role of thylakoid AQPs in plant photosynthesis, which needs to be addressed in the future.

Excess light absorption by the thylakoid results in thermal dissipation in antenna and the formation of reactive oxygen species (ROS), such as superoxide anion radical, O_2_^•−^, and hydrogen peroxide, H_2_O_2_, which were detected in isolated thylakoids [27,28]. Even though it was known that ROS are damaging to the cell, they also play a major role in cellular signaling pathways, especially in the case of H_2_O_2_ [29]. Numerous studies demonstrated that AQPs facilitated transmembrane diffusion of H_2_O_2_ with a heterologous expression system in yeast [30,31,32], but the diffusion of H_2_O_2_ through the chloroplast envelope is still a matter of debate and needs to be studied further. It was observed that outside chloroplasts, the Resorufin fluorescence, which was a probe for detecting H_2_O_2_ as the reaction product of Amplex Red and H_2_O_2_, was suppressed by 60% in the presence of an aquaporin inhibitor, indicating that H_2_O_2_ can diffuse through the chloroplast envelope aquaporins [28].

### 2.2. CO_2_ Transport Facilitator

**Figure 2:**
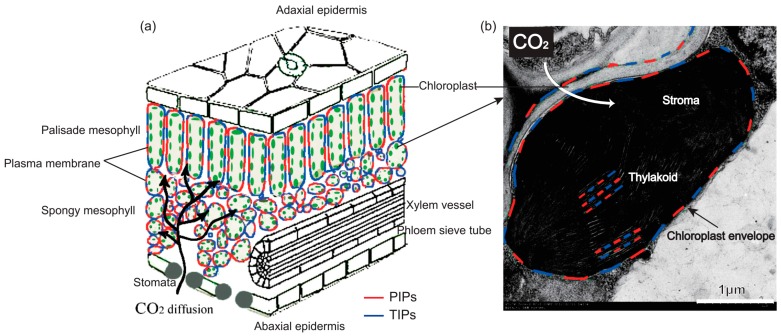
Pathway of CO_2_ diffusion to substomatal internal cavities (**a**) and from there to chloroplasts (**b**). The presence of PIPs (red) and TIPs (blue) in the plasma membrane and chloroplast envelope facilitated CO_2_ transport, and PIPs and TIPs located in the thylakoid implied their role in mediating H_2_O transport, which are reviewed in Section 2.2. The bar indicates 1 μm.

For CO_2_ assimilation in C3 leaves, CO_2_ needs to diffuse from the foliage surface to substomatal internal cavities and to within chloroplasts, with resistance along the diffusion path measured as the CO_2_ transfer conductance in the stomata (*g_s_*) and mesophyll cell (*g_m_*), respectively (Figure 2). CO_2_ in the chloroplast (*C_c_*) is catalyzed by the primary CO_2_-fixing enzyme, ribulose 1,5-bisphosphate carboxylase/oxygenase (Rubisco), which is a bifunctional enzyme that both CO_2_ and O_2_ compete over for reaction with ribulose-1,5-disphosphate (RuBP). The oxygenation of RuBP leads to carbon loss and is energy-consuming, so it is essential to keep the CO_2_ concentration in chloroplasts as high as possible. It is proposed that the photosynthesis rate (*P_n_*) in C3 plants is mainly limited by Rubisco carboxylation activity in full sunlight under the current atmospheric CO_2_ concentration, which emphasizes the critical role of both *g_s_* and *g_m_* in mediating carbon metabolism [33]. A large number of studies have demonstrated the positive relationship between CO_2_ diffusion conductance and *P_n_* and proved the vital role of *g_s_* and *g_m_* in shaping CO_2_ fixation [34,35,36,37,38,39].

**Figure 3:**
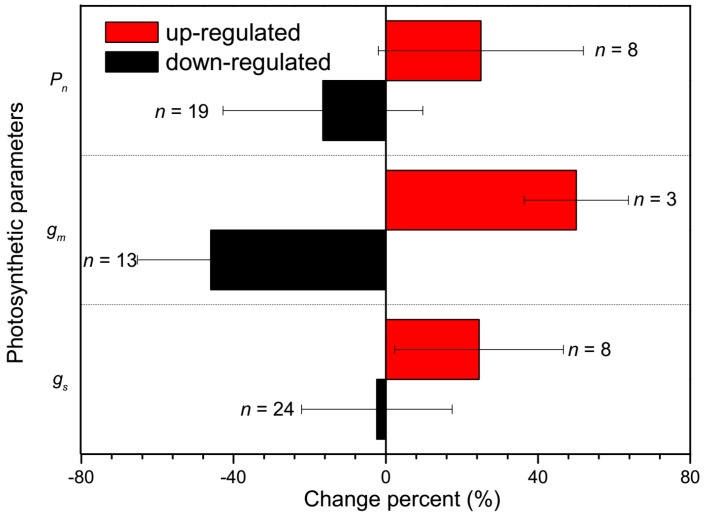
The change in photosynthesis rate (*P_n_*), mesophyll conductance (*g_m_*), and stomatal conductance (*g_s_*) in up-regulated AQP (in red) and down-regulated AQP (in black) plants compared with wild type plants. The data were collected from Flexas et al. [7], Uehlein et al. [25], Uehlein et al. [42], Heckwolf et al. [43], Hanba et al. [45], Secchi and Zwieniecki [50], Tsuchihira et al. [51], Kawase et al. [52], Sade et al. [53] and Li et al. [54]. The sample number of each parameter was indicated by *n*.

Expression of *AQP1* in *Xenopus oocytes* in the presence of carbonic anhydrase significantly increased CO_2_ permeability of oocyte membranes, which demonstrated that *AQP1* acted as a CO_2_ channel [40]. Further results revealed that *C_c_* decreased by 60–70% after the addition of an AQP inhibitor, HgCl_2_, which implied that HgCl_2_-sensitive AQPs facilitated CO_2_ uptake across the plasma membrane of the mesophyll cell [41]. Direct evidence was found in 2003, when Uehlein et al. [42] firstly demonstrated a CO_2_ permeability comparable to that of the human *AQP1* for the tobacco plasma membrane aquaporin *NtAQP1* when expressed both heterologously in *Xenopus oocytes* and in a plant system, in which *g_s_* was evidently increased in *NtAQP1* overexpression. Further, Flexas et al. [7] demonstrated that the difference in *g_m_* explained the differences in net photosynthesis between tobacco *NtAQP1* antisense and overexpressed tobacco plants by analyzing the chlorophyll fluorescence and on-line ^13^C discrimination. Even though *NtAQP1* belonged to the so-called PIP1 which is located in the plasma membrane, it was also reported located in the chloroplast envelope (Figure 2), and the decreased *NtAQP1* expression lowered the CO_2_ permeability of the inner chloroplast membrane [25]. The role of AQPs to act as CO_2_ transport facilitators was also suggested in *Arabidopsis thaliana* [43,44], *Hordeum vulgare* [45,46,47], *Oryza sativa* [39], *Zea mays* [48], *Terfezia claveryi* [49], and *Populus tremula × alba* [50], all these studies clearly demonstrated the beneficial role of AQPs in mediating CO_2_ conductance. We recalculated the published data and compared the effects of AQPs on *g_s_* and *g_m_* (Figure 3). The results showed that the contribution of AQP expression on *g_m_* was significantly higher than that on *g_s_*, which was increased by 25% in the up-regulated plants compared with the wild type plant, while no significant decrease in *g_s_* of down-regulated plants was observed. In contrast, the *g_m_* was increased by 50% and decreased by 46% in up- and down-regulated AQP expression plants, respectively; however, no significant difference in *P_n_* was concluded between both up- and down-regulated plants and wild type plants (Figure 3). This result suggested that the *g_m_* was not always the limiting factor for *P_n_*, or other genes compensated for the function of particular absent genes.

## 3. Role of AQPs in Regulating Nitrogen Status

**Figure 4:**
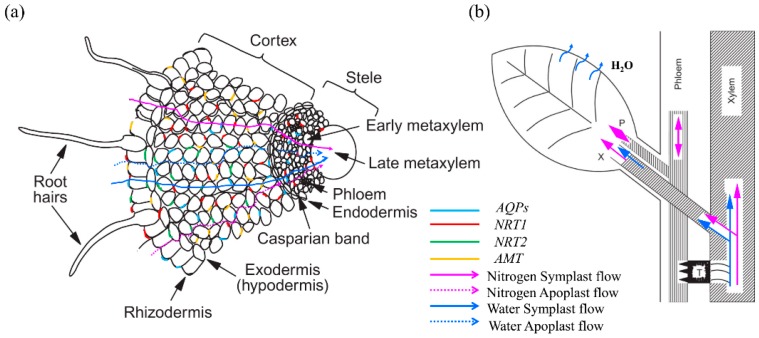
Diagrammatic illustration of the water and nitrogen uptake and transport from the soil through the soil to the root (**a**) and further to the shoot (**b**). Both water and nitrogen can flow either via apoplast (dotted arrows) or symplast (solid arrows) pathways. *NRT1* transporter (in red) is responsible for both NO_3_^−^ uptake and radial and long-distance transport, while *NRT2* transporter (in green) is only involved in NO_3_^−^ uptake. For NH_4_^+^, the *AMT* family represents the major entry pathway for root NH_4_^+^ uptake, but the transporters involved in NH_4_^+^ xylem loading in the root and unloading in the shoot are unknown. For water, it can flow either via the apoplast or through AQPs via the symplast, and further be transported to the shoot where it is lost to the atmosphere by transpiration from leaves.

Ammonium (NH_4_^+^) and nitrate (NO_3_^−^) are major inorganic N sources that can be used by plants. Soil NH_4_^+^ and NO_3_^−^ are carried towards the root by bulk flow; *NRT* and *AMT* families are involved in the uptake of NO_3_^−^ and NH_4_^+^ from soil, after which they are transported either via apoplast or symplast pathways to the stele (Figure 4a) [55,56,57]. Once NO_3_^−^ and NH_4_^+^ are loaded into the xylem—a process which is regulated by *NRT* and unknown factors, respectively—N is transported to the shoot with the transpiration stream (Figure 4b). Therefore, water flow through plants affects N acquisition and delivery from the soil, and adequate water supply is critical for N movement in the plants. Since root hydraulic conductivity and xylem–mesophyll water transport are largely dependent on AQPs in the root and vascular bundle-sheath cells [58,59], there is no doubt that N uptake and transport in plants are largely regulated by AQPs in their function in regulating water flow. Links between AQPs, water status, and N uptake or transport in plants have been widely proposed, with evidence showing that AQP overexpression positively affects the transpiration rate (*T_r_*) and total N uptake, while the addition of AQP inhibitor induces decreased *T_r_* and xylem sap flow rate, resulting in impeded N mass flow in the soil solution to roots [60,61,62,63].

AMT transporters represent the major entry pathway for root NH_4_^+^ uptake (Figure 4), and the expression of *AtTIP2;1*, *AtTIP2;3*, *ZmTIP1;1*, *TaTIP2;1*, and *ZmTIP1;2* in heterogenous systems are also demonstrated facilitating NH_3_ and/or NH_4_^+^ transport (Table 1) [64,65,66]. Clear evidence shows that *TaTIP2;2* was a separate NH_3_ pore rather than being conductive for NH_4_^+^ when expressed in yeast [67]. After transferring from NO_3_^−^ to NH_4_^+^ nutrition, expression of *AtTIP2;1* and *AtTIP2;3* were up-regulated and the NH_4_^+^ concentration was increased in *Arabidopsis thaliana* roots, while in transgenic *Arabidopsis thaliana* plants overexpressing *AtTIP2;1*, the NH_4_^+^ accumulation in roots after NH_4_^+^ supply did not show any alteration [64]. The explanation for this phenomenon is that (1) the posttranscriptional down-regulation of *AtTIP2;1* offset the increased expression of *AtTIP2;1*; (2) the NH_4_^+^ concentration in the roots failed to reflect the condition in the vacuole; and (3) *AtTIP2;1*-overexpression could not effectively increase NH_3_ transport if there was already a high density of TIPs in the wild-type plants’ tonoplast. Since excess uptake of ammonium is harmful to plant cells, ammonium uptake across the root plasma membrane has to be tightly regulated [64]; different mechanisms have been proved to be associated with ammonium toxicity tolerance. Upon elevated ammonium supply, *AMT1;1* and *AMT1;3* were inactivated either by phosphorylation of the C-terminal domain or by protein clustering and endocytosis, thereby shutting off ammonium acquisition [68,69,70,71]. Physiological experiments have indicated that ammonium is also exported from the cytoplasm into the apoplasm or into the vacuole, which implies that AQPs also participate in the alleviation of ammonium toxicity by removing excess NH_3_ and NH_4_^+^ from the cytoplasm and/or by compartmenting NH_3_/NH_4_^+^ in the vacuole. Recently, Coskun et al. [72] proposed that NH_3_ rather than NH_4_^+^ transport is responsible for the futile transmembrane cycling under NH_3_/NH_4_^+^ toxicity in plant roots, and this process is considered as a downstream target of intracellular ammonium sensing by the tonoplast-localized receptor-like kinase CAP1 [73]. In conclusion, both AMT and AQPs contributed to the NH_3_/NH_4_^+^ transport and homeostasis in plants by controlling their acquisition and exportation or compartmentation.

Urea is a main N fertilizer used in agricultural production and has been demonstrated to cross the bio-membrane through AQPs (Table 1), as *NtAQP1* had been demonstrated permeable for urea in the *Xenopus oocytes* [74]. *ZmPIP1-5b*, which was isolated from maize hybrid F2F7, also exhibited urea transport activity in the *Xenopus oocytes* system [75]. Except for PIPs, nodulin-26-like intrinsic proteins (NIPs) also showed urea permeability (Table 1). Yeast mutant *vdur3*, which was unable to grow on media containing low concentrations of urea as the sole nitrogen source, regained the capacity to grow on low urea when complemented by *CpNIP1* [76]. Similar results were also obtained as the performance of *ZmNIP2;1*, *ZmNIP2;4*, *AtNIP5;1*, *AtNIP6;1*, and *CsNIP2;1*, which showed urea transport ability when expressed in *Xenopus oocytes* or yeast [77,78,79]. TIPs probably function in transporting urea more than as water channels (Table 1): In recent years, by using stopped-flow spectrofluorimetry, tobacco tonoplast vesicles (*NtTIPa*) were found with urea permeabilities [80], and by using complementary assay, *AtTIP1;1*, *AtTIP1;2*, *AtTIP2;1*, *AtTIP4;1*, and *ZmTIP4;4* conferred the growth of a urea uptake-defective yeast mutant [76,77]. Moreover, *AtTIP1;3* and *AtTIP5;1* were suggested as the water and urea channels in mature pollen and responsible for nitrogen remobilization in *Arabidopsis* [81,82]. It is noteworthy that the majority of the research was conducted in heterologous expression systems such as yeast and *Xenopus oocytes* to clarify the role of AQPs in facilitating urea transport rather than in the plant cell, which needs to be studied in future.

## 4. Responses of AQPs to Nitrogen Supply

**Figure 5:**
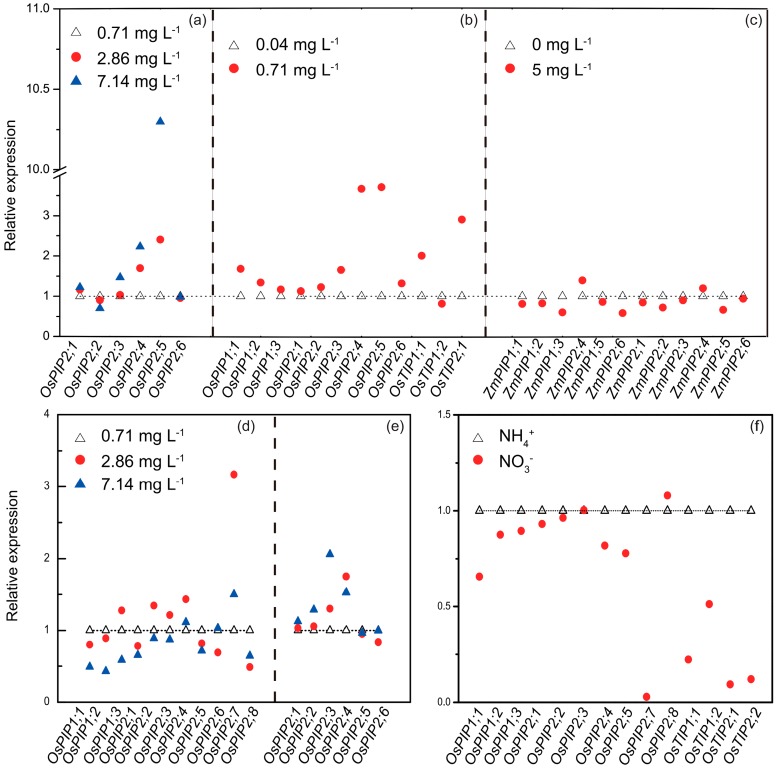
Effect of different nitrogen supply amount (**a**–**e**) and nitrogen forms (**f**) on AQP expression in rice root (**a**,**b**,**f**), rice leaf (**d**,**e**), and maize root (**c**). Relative gene expression was presented as the fold change compared to the expression under low N supply (**a**–**e**) and NO_3_^−^ supply (**f**). The response of leaf AQPs to N supply was shown in different rice cultivars, “Shanyou 63” (hybrid *indica* China, **d**) and “Yangdao 6” (conventional *indica* China, **e**). AQP expression in the rice was determined after treatment for 2 weeks (**a**,**d**–**f**) or 24 h (**b**), and the AQP expression in the maize was averaged from the expression determined after the treatments for 0.5 h, 1 h, 2 h, and 4 h (**c**). The data were extracted from Ren et al. [85] (**a**), Wang et al. [88] (**b**), Gorska and Zwieniecki [86] (**c**), Ding et al. [39] and Ren et al. [85] (**d**), Ren et al. [85] (**e**), Wang et al. [93] and Ding et al. [94] (**f**).

Different AQP responses have been observed under various nitrogen availabilities. A lack of Hg-sensitivity in plasma membranes from nitrogen-deprived roots evidenced a lowering in the AQP activity and/or the density [84]. Recently, Ren et al. [85] claimed that, relative to low nitrogen, high nitrogen supply enhanced AQP expression in rice roots (Figure 5a). It is noteworthy that the response of AQPs to the nitrogen supply is dependent on treatment duration. In maize, no changes in *ZmPIP1s* and *ZmPIP2s* gene expression were found after NO_3_^−^ addition for 4 h to the N-starved treatments [86] (Figure 5c), and after treatment with NO_3_^−^ for 20 min, Wang et al. [87] detected that only *NIP2.1* was suppressed and others were kept constant. In contrast, Wang et al. [88] showed that root aquaporin genes were significantly up-regulated after being nitrogen-induced for 24 h (Figure 5b), including *OsPIP1;1*, *OsPIP2;2*, *OsPIP2;3*, *OsPIP2;4*, *OsPIP2;5*, *OsPIP2;6*, *OsTIP1;1*, and *OsTIP2;1* [89]. Similarly, di Pietro et al. [90] found decreased root *PIP2;1*, *PIP2;2*, *PIP2;4*, *PIP1;2*, and *PIP1;3* expression in response to nitrogen starvation for 6 days in *Arabidopsis* [84,91].

In the case of leaves, Ding et al. [39] demonstrated the down-regulation of *OsPIP1s* gene expression under high nitrogen supply compared with low N supply (Figure 5d); the decreased PIP1 clades expression, which is predominantly responsible for CO_2_ transport, induced decreased CO_2_ transportation and relatively lower CO_2_ concentration in the chloroplast (*C_c_*). Eventually, under high N supply, the leaf AQPs were unable to sustain the C/N balance as the down-regulated AQPs, and eventually resulted in decreased photosynthetic nitrogen use efficiency. However, the intrinsic mechanisms of the decreased leaf AQP expression under high N supply remains unclear. Besides this, Ding et al. [39] and Ren et al. [85] also concluded that most of the *OsPIP2s* gene expression, such as *OsPIP2;1*, *OsPIP2;2*, *OsPIP2;3*, *OsPIP2;4*, *OsPIP2;5*, and *OsPIP2;8*, was down-regulated under a high nitrogen supply compared with under a low or intermediate nitrogen supply in hybrid rice (Figure 5d); however, the expression of these genes was up-regulated with increasing nitrogen supply in conventional rice (Figure 5e), implying that the responses of AQPs to nitrogen supply are dependent on plant varieties [83].

AQPs not only respond to N availability, but also to N forms, including ammonium (NH_4_^+^) and nitrate (NO_3_^−^) (Figure 5e). AQP expression was lower under NH_4_^+^ supply than under nitrate supply in *Phaseolus vulgaris* L. [92]; on the contrary, Wang et al. [93] and Ding et al. [94] concluded significant up-regulated AQP expression under NH_4_^+^ nutrition compared with NO_3_^−^ nutrition in *Oryza sativa* L. (Figure 5e). This difference likely results from the preference to N sources in various plant species. One important reason that contributes to the response of AQPs to N forms is phosphorylation modification, since Engelsberger and Schulze [68] found a more sensitive response by AQP phosphorylation to NH_4_^+^ than to NO_3_^−^ nutrition.

## 5. Conclusions and Future Perspective

AQPs functions as water and CO_2_ transport facilitators in the chloroplast, and are essential for the successful operation of photosynthesis. The N status in plants is influenced by AQPs either through regulating water flow or by facilitating NH_4_^+^/NH_3_ and urea transport, and AQPs have also been proposed to associate with NH_4_^+^ toxicity alleviation by means of vacuole compartmentation. In turn, both N availability and forms have great impacts on AQP expression. It is important to address the following issues in the coming future: (1) experimental evidence should be unfolded to directly clarify the role of AQPs in electron transport, as the location of AQPs in the thylakoid and chloroplast inner membrane have been confirmed; (2) the role of AQPs in mediating N metabolism in plants should be focused on, e.g., the response of plant growth to NH_4_^+^ nutrition in different N forms and the preference of the plant species, the function of AQPs in alleviating NH_4_^+^ toxicity, and related mechanisms about the effect of AQPs on ammonia volatilization from plants; (3) the role of AQPs in mediating the C/N balance should be concentrated on the basis of the determinate function of AQPs in regulating C and N status.

## Figures and Tables

**Table 1:** Properties of aquaporins related to nitrogen nutrition.

Substrate	Family	Name	Expression System	References
NH_3_	*TIPs*	*AtTIP2;1*	yeast	Loque et al. [64]
		*AtTIP2;3*	yeast	Loque et al. [64]
		*ZmTIP1;1*	yeast	Barzana et al. [65]
		*ZmTIP1;2*	yeast	Barzana et al. [65]
		*TaTIP2;1*	*Xenopus oocytes*	Holm et al. [66]
		*TaTIP2;2*	yeast	Bertl and Kaldenhoff [67]
Urea	*PIPs*	*NtAQP1*	*Xenopus oocytes*	Eckert et al. [74]
		*ZmPIP1;5*	*Xenopus oocytes*	Gaspar [75]
	*NIPs*	*CpNIP1*	yeast	Klebl et al. [76]
		*ZmNIP2;1*	yeast	Gu et al. [77]
		*ZmNIP2;4*	yeast	Gu et al. [77]
		*AtNIP5;1*	*Xenopus oocytes*	Wallace and Roberts [78]
		*AtNIP6;1*	*Xenopus oocytes*	Wallace and Roberts [78]
		*CsNIP2;1*	yeast	Zhang et al. [79]
	*TIPs*	*NtTIPa*	*Xenopus oocytes*	Gerbeau et al. [80]
		*AtTIP1;1*	yeast	Liu [83]
		*AtTIP1;2*	yeast	Liu [83]
		*AtTIP2;1*	yeast	Liu [83]
		*AtTIP4;1*	yeast	Liu [83]
		*AtTIP1;3*	*Xenopus oocytes*	Soto et al. [81]
		*AtTIP5;1*	*Xenopus oocytes*	Soto et al. [82]
		*ZmTIP4;4*	yeast	Gu et al. [77]
